# New Insight for Surface Chemistries in Ultra-thin Self-assembled Monolayers Modified High-voltage Spinel Cathodes

**DOI:** 10.1038/s41598-018-30135-z

**Published:** 2018-08-06

**Authors:** Dae-wook Kim, Shuhei Uchida, Hiromasa Shiiba, Nobuyuki Zettsu, Katsuya Teshima

**Affiliations:** 1 Department of Materials Chemistry, Faculty of Engineering, Shinshu University, 4-17-1 Wakasato, Nagano, 380-8553 Japan; 2 Center for Energy & Environmental Science, Shinshu University, 4-17-1 Wakasato, Nagano, 380-8553 Japan

## Abstract

The electrochemical properties of the interface between the spinel LiNi_0.5_Mn_1.5_O_4-*δ*_ (LNMO_4-*δ*_) cathodes and ethylene carbonate−dimethyl carbonate (EC-DMC) electrolyte containing 1 M of LiPF_6_ have been investigated to achieve high-voltage durability of LNMO_4-*δ*_/graphite full cells. Coating the LNMO_4-*δ*_ crystal surface by a fluoroalkylsilane self-assembled monolayer with a thickness below 2 nm resulted in a capacity retention of 94% after 100 cycles at a rate of 1 C and suppression of capacity fading for both the cathode and anode of the full cell. The observed effect is likely caused by the inhibited oxidative decomposition of EC−DMC electrolyte and vinylene carbonate (VC) species at the LNMO_4-*δ*_ crystal surface and formation of a stable VC solid electrolyte interface near the anode. Moreover, the results obtained via photoelectron spectroscopy and density-functional calculations revealed that the increase in the work function of the LNMO_4-*δ*_ crystal surface due to the formation of Si−O−Mn species primary contributed to the inhibition of the oxidative decomposition of the electrolyte and VC molecules at the cathode/electrolyte interface.

## Introduction

During recent years, the industrial demand for high-power energy storage devices with large energy densities has been continuously growing. Lithium-ion batteries (LIBs) are very promising energy storage systems for electric vehicles that require relatively high energy densities. In particular, increasing the operational voltage corresponding to the redox potential for the intercalation reaction of Li ions at the cathode above 4.45 V (vs Li^+^/Li) represents one of the most serious issues for high-energy density LIBs. Since lowering the level of the d band occupied by the electrons of the transition metal component of the LIB cathode noticeably increases its redox potential, layered materials with high Ni contents (such as NCM811 and NCA) have attracted significant attention. Among these cathode materials, high-voltage spinels, such as LiNi_0.5_Mn_1.5_O_4_ (LNMO) and related compounds, have been also considered for this purpose because of the absence of Co elements, ability to be easily handled in air, thermal stability in the presence of O_2_ gas at high temperatures, and existence of a robust spinel framework after full delithiation^1–4^. However, their high operating voltages (>4.4 V vs. Li^+^/Li) commonly result in the oxidative decomposition of the electrolyte at the delithiated LNMO electrode surface, which subsequently promotes various side reactions, including Mn^2+^ elution, the formation of metal fluorides, and solid electrolyte interface (SEI) deposition^5–9^. In particular, the reliability of the high-voltage spinel-based LIBs is negatively affected by the migration of eluted Mn ions from the cathode to the anode. It can be disturbed intercalation for Li ion, which renders consuming active Li (formation of thicker SEI) and consequently, irreversible capacity loss occurs^10–12^.

Many researchers have attempted to mitigate the observed capacity fading of LNMO/graphite cells through the reduction of the direct contact area of the cathode with the electrolyte as well as via the passivation of transitional metal-metal and oxygen-oxygen bonds at the cathode surface by coating it with inorganic particles (such as ZrP_2_O_7_, ZnAl_2_O_4_, LiBOB, TiO_2_, Al_2_O_3_, SiO_2_, and graphene oxide) as well as by adding polymeric electrolytes and organic modifiers to the electrolyte^13–21^. Various fundamental studies have been performed to investigate the effects of the surface coating and electrolyte modification; however, none of them led to a considerable performance enhancement of high-voltage spinel cathode-based battery cells.

Recently, we have found that ultra-thin fluoroalkylsilane (FAS) self-assembled monolayer (SAM) coatings can be used as surface modifiers for the enhancement of high-voltage durability of LiNi_0.5_Mn_1.5_O_4-*δ*_ (LNMO_4-*δ*_) cathodes immersed in the ethylene carbonate–dimethyl carbonate (EC–DMC) electrolyte (containing 1 M of LiPF_6_) of Li half-cells^22^. The analysis of their kinetic parameters revealed that the SAM coatings with thicknesses below 2 nm did not apparently increase the charge transfer resistance of the LNMO_4-*δ*_/electrolyte interface and degrade rate capability of the cells after cycling. Although the decrease in the effective specific surface area of the cathode that minimized its direct contact with the electrolyte contributed to the observed enhancement of the capacity retention, the effects produced by the SAM coatings on the electrochemical properties of high-voltage spinel systems have not been examined in sufficient detail. Hence, the systematic studies of the cathode surface view point of electric structures used by various surface chemical states analysis techniques based on photoelectron spectroscopies for SAM-coated electrodes are essential for elucidating the suppression of the capacity fading mechanism on the SAM-coated high-voltage spinel electrodes.

In this work, the effects produced by the SAM coatings of LNMO_4-*δ*_ cathode materials were investigated using various electrochemical and surface analysis techniques combined with density-functional theory (DFT) computations. The obtained results can provide new directions for designing LIBs based on high-voltage spinel systems with superior electrochemical performance.

## Results

The effect of the SAM coatings on the chemical processes that occur at the LNMO electrode/electrolyte interface is strongly related to the high-voltage durability and kinetics parameters of the intercalation/deintercalation reactions. In this work, ultra-thin monolayers containing FAS and octadecylsilane (ODS) species have been deposited on the LNMO electrodes using the vapor-phase method, which usually results in the formation of a dense monolayer due to the Langmuir-type adsorption. The Si2p/Mn2p_3/2_ peak area ratios of the FAS17 and ODS components drastically changed with increasing treatment time and became constant after 15 h for FAS17 and 36 h for ODS, as shown in Figure S1. If a multilayer of inhomogeneous aggregates is formed, the relative intensities of these peaks would linearly increase with time, indicating that the LiNi_0.5_Mn_1.5_O_4-*δ*_ surface is fully covered with a SAM of organosilane molecules according to the Langmuir absorption model. The Takai research group has previously reported that the thicknesses of the FAS17 and ODS layers formed on a SiO_2_/Si substrate were 1.34 nm and 1.4 nm^23^, respectively, which was in good agreement with the results of angle-resolved XPS and semi-empirical analyses^22^.

**Figure 1:**
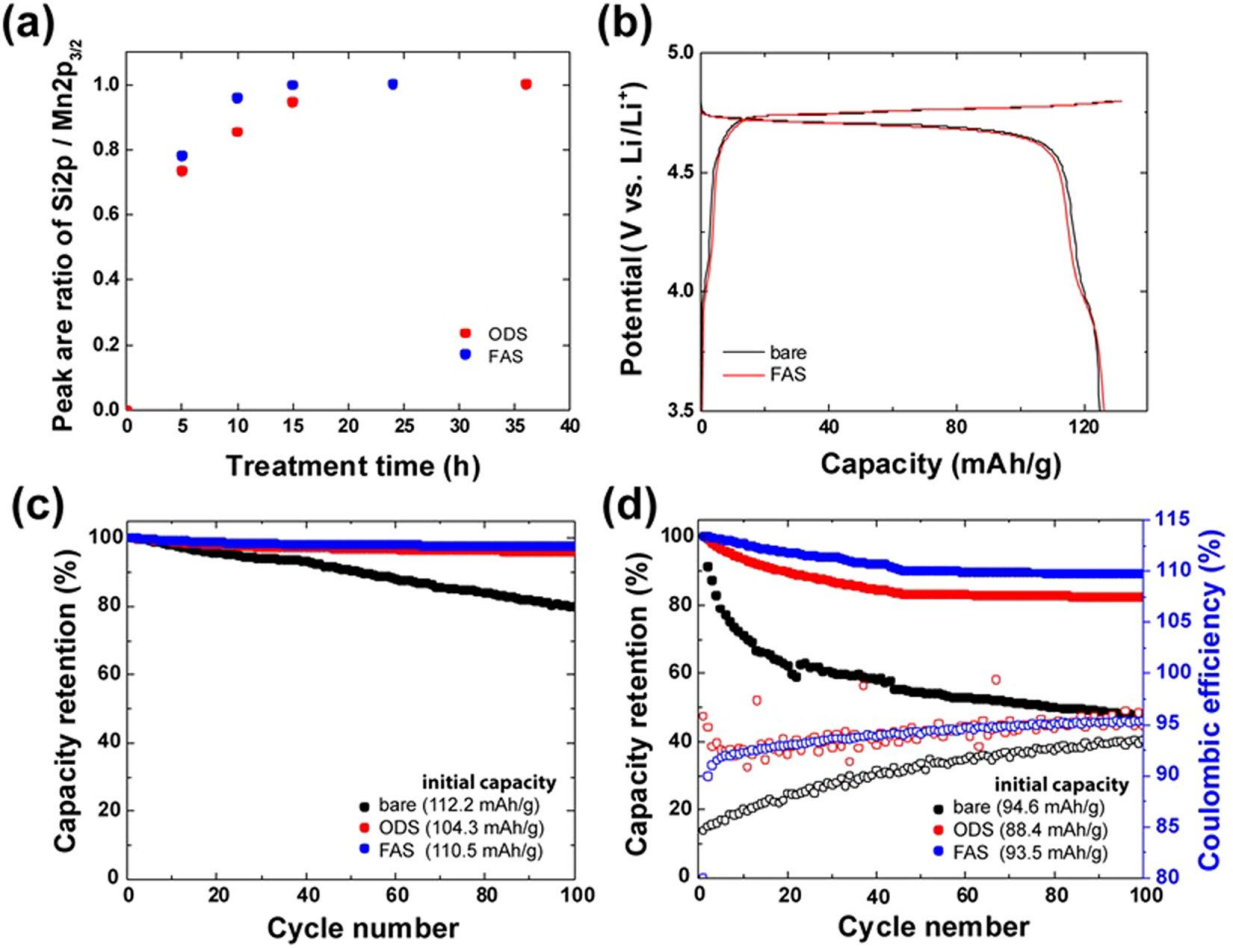
(**a**) Si 2p/Mn 2p_3/2_ relative peak areas of the SAM-coated LNMO_4-*δ*_ crystals plotted as functions of coating time. (**b**) Charge–discharge profiles of the LNMO_4-*δ*_/graphite cell operated at a rate of 0.2 C. Capacity retention of the (**c**) LNMO_4-*δ*_/Li and (**d**) LNMO_4-*δ*_/graphite cells at a rate of 1 C for 100 cycles.

The galvanostatic charge–discharge curves obtained at a current density of 0.2 C for the bare and SAM-coated electrodes are shown in Fig. 1b. No significant differences were observed between the specific capacities, Coulombic efficiencies, and curve profiles of these systems. The obtained results suggest that the formation of FAS and ODS monolayers on the electrode surfaces did not disturb the transport of Li ions between the electrode and electrolyte at a low current density corresponding to the charge/discharge rate of 0.2 C.

To investigate the impact of the SAM coatings on the electrolyte oxidation, which directly affected the cycling performance of the cell, cycling tests were performed at room temperature and a rate of 1 C for 100 cycles (Fig. 1c). The cycle tests were demonstrated at room temperature in order to simplify complex reaction appeared at the electrode surface through the removal of disturbances and unexpected parasitic reactions at high temperature. During half-cell testing, the capacity retentions of the FAS- and ODS-coated samples were 97 and 96%, respectively, while the bare LNMO electrode exhibited a capacity retention of 78%. The bare reference electrodes demonstrated here poorly performed as compared to other data presented in literatures. This is because that the LNMO crystals prepared from molten KCl, resulting in smaller diameter (D_50_ = 1.01 μm) with higher surface area (0.32 m^2^·g^−1^), as comparing to that of solid-state synthesis. Furthermore, the crystals included oxygen deficient, leading to form Jahn-Teller Mn^3+^. Those power characteristics might promote the capacity fading kinetically due to oxidative decomposition of liquid electrolytes. Furthermore, we used higher tap density electrode of >3.0 g/cc due to achieve high energy density LIB.

In contrast, significant capacity fading was observed for the bare LNMO/graphite full-cell. The FAS- and ODS-coated electrodes of these cells demonstrate relatively small capacity fading corresponding to 90 and 84% of the initial capacity after 100 cycles, respectively, while the magnitude obtained for the bare LNMO/graphite full-cell was 50%. These results imply that the surface coatings inhibit the parasitic reactions occurring at the LNMO cathode and graphite anode interfaces of the cells. Further, galvanostatic charge-discharge tests were performed using a three-electrode cell system, which was capable of separately evaluating the charge and discharge characteristics of the LNMO and graphite electrodes (Figure S1). For both electrodes, the shape of the charge-discharge profile was related to their capacity fading properties. As compared to the control sample containing a bare LNMO_4-*δ*_ electrode as the cathode, the full cells with the SAM-coated LNMO_4-*δ*_ electrodes exhibited significantly better cycling performance. Moreover, the cells containing bare LNMO_4-*δ*_/graphite electrodes exhibited a poor Coulombic efficiency of around 92% after 100 cycles, while the cells composed of the FAS17- and ODS-coated LNMO_4-*δ*_/graphite electrodes were presented 95 and 96%, respectively (Fig. 1d). It is suggesting that the SAM coatings clearly mitigated the effects of the end-point capacity slippage (a high Coulombic efficiency close to 1.000 indicates a negligible number of parasitic reactions occurring inside the Li-ion cell). Furthermore, the capacity of the LNMO_4-*δ*_ electrode deteriorated with respect to that of the graphite anode, which significantly contributed to the fading of the capacity retention of the studied full cells.

**Figure 2:**
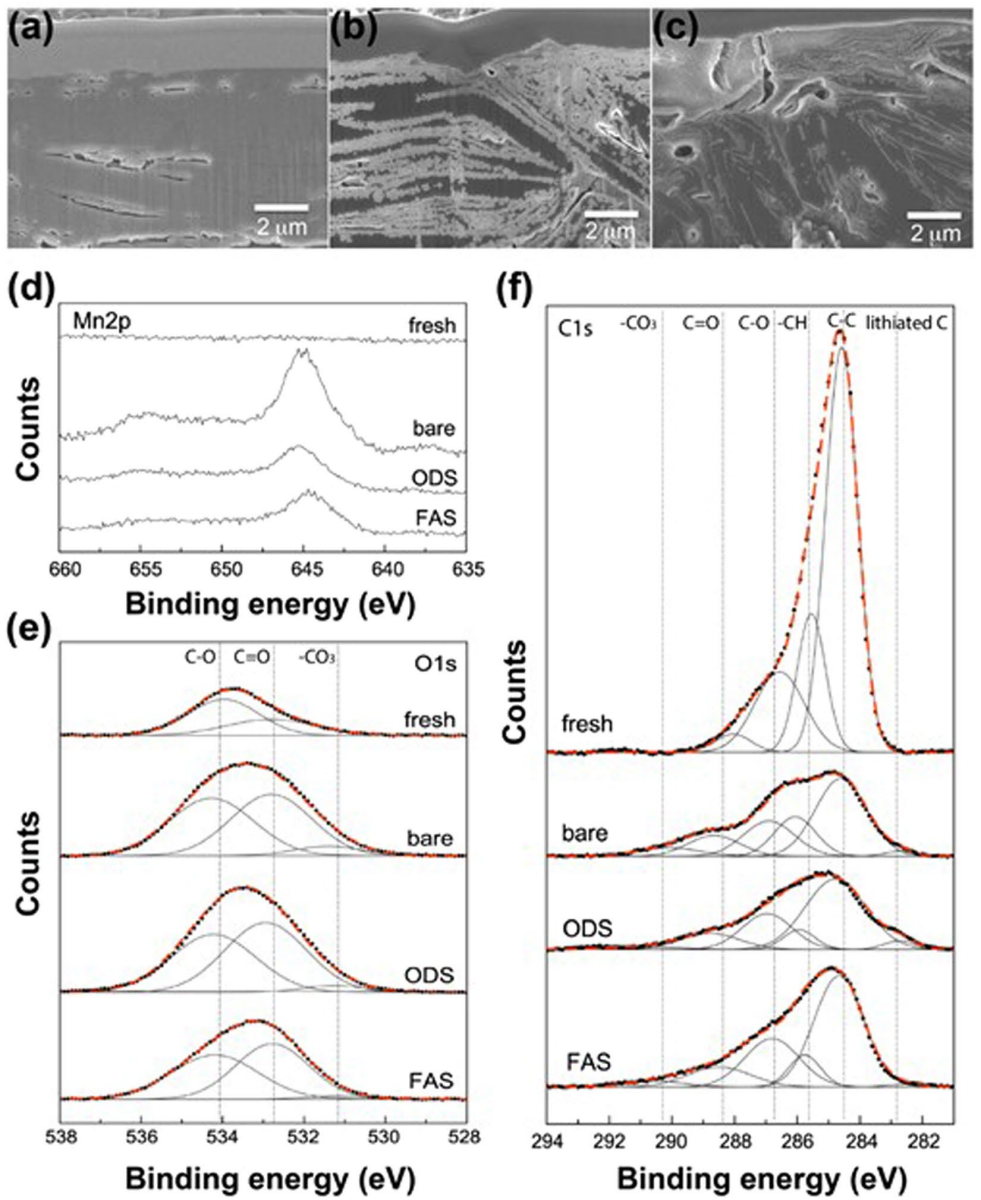
Impact of the SAM coatings on the chemical reaction at the graphite electrode surface. Cross-sectional FE−SEM images of the (**a**) as-prepared graphite electrode, (**b**) graphite electrode coupled with bare LNMO_4-*δ*_ cathode and (**c**) the FAS-coated LNMO_4-*δ*_ cathode. (**d**) Mn 2p, (**e**) O 1s, and (**f**) C 1s XPS core level spectra of the graphite electrode after 100 cycles.

FE-SEM observations and XPS analysis of the graphite anodes extracted from the full cells after 100 cycles and carefully washed with DMC were performed to elucidate the mechanism of the chemical reaction occurring at the graphite anode surface. Figure 2a–c shows the morphological changes of the graphite anodes evaluated via the cross-sectional FE-SEM observation. Their morphologies were found to be significantly dependent on the SAM coating on the LNMO_4-*δ*_ cathode. A thick SEI layer was excessively deposited on the graphite anode surface coupled with the bare LNMO_4-*δ*_ cathode as compared to that of the FAS17-coated LNMO_4-*δ*_/graphite cell. These morphological differences strongly suggests that SEI layer have different chemical compositions depending on the SAM composition. The results of energy-dispersive X-ray spectroscopy (EDS) analysis revealed that the formed SEI layer on the anode surface was primary composed of Li, F, P, and C elements, which likely originated from LiF, Li_x_PF_y_O_z_, and CH_x_CF_y_ species (Figure S2). X-ray photoelectron spectroscopy (XPS) was further performed in order to evaluate the SEI structures.

The XPS Mn 2p core level spectra of the graphite anodes are shown in Fig. 2d. While the positions of the XPS peaks exhibited no apparent dependences on the SAM composition, their relative intensities significantly varied. The graphite anode coupled with the bare LNMO_4-*δ*_ cathode produced a remarkably intense signal, which could be attributed to the transport and deposition of dissolved Mn ions from the cathode surface. It is commonly accepted that the mechanism for Mn and Ni dissolution involves a disproportionation reaction of Mn^3+^ to Mn^2+^ and Mn^4+^. Very recently, Shkrob *et al*.^24^ suggested that Mn^2+^ species were containing manganese acetate. Cabana *et al*.^25^ demonstrated that the impedance of the graphite anode was directly proportional to the concentration of Mn^2+^ species in the SEI layer. There is no doubt that Mn^2+^ species originate from the Mn-based oxide positive electrode. Therefore, the obtained XPS Mn 2p spectra clearly suggest that the SAM coating deposited onto the cathode surface is able to mitigate the effects of capacity fading and related charge end-point capacity slippage caused by the reduction of Mn ions traveled from the LNMO_4-*δ*_ cathode.

The O 1s core-level XPS spectra of the graphite electrodes are shown in Fig. 2e. Their asymmetrically broad peaks can be deconvoluted into three components corresponding to ethers (C–O), esters (C=O), and carbonates (–CO_3_). Both the C–O and C=O components of the fresh graphite anode resulted from the binders utilized during the manufacturing of graphite electrodes. It should be noted that the XPS carbonate peaks of the SAM-coated LNMO_4-*δ*_ cathode/graphite cells were weaker than that of the bare LNMO/graphite cell. Furthermore, a negligible amount of carbonates was observed on the anode surface detached from the FAS17-LNMO cathode/graphite cell. Since these carbonates likely correspond to the Li_2_CO_3_ and Li_2_C_2_O_4_ species generated by of the reaction of CO_2_ with the products of the oxidative decomposition of the electrolytic solution that occurs at the LNMO_4-δ_ cathode surface, the self-assembled FAS17 monolayer exhibits a significant impact on the suppression of the oxidative decomposition of the electrolyte.

**Figure 3:**
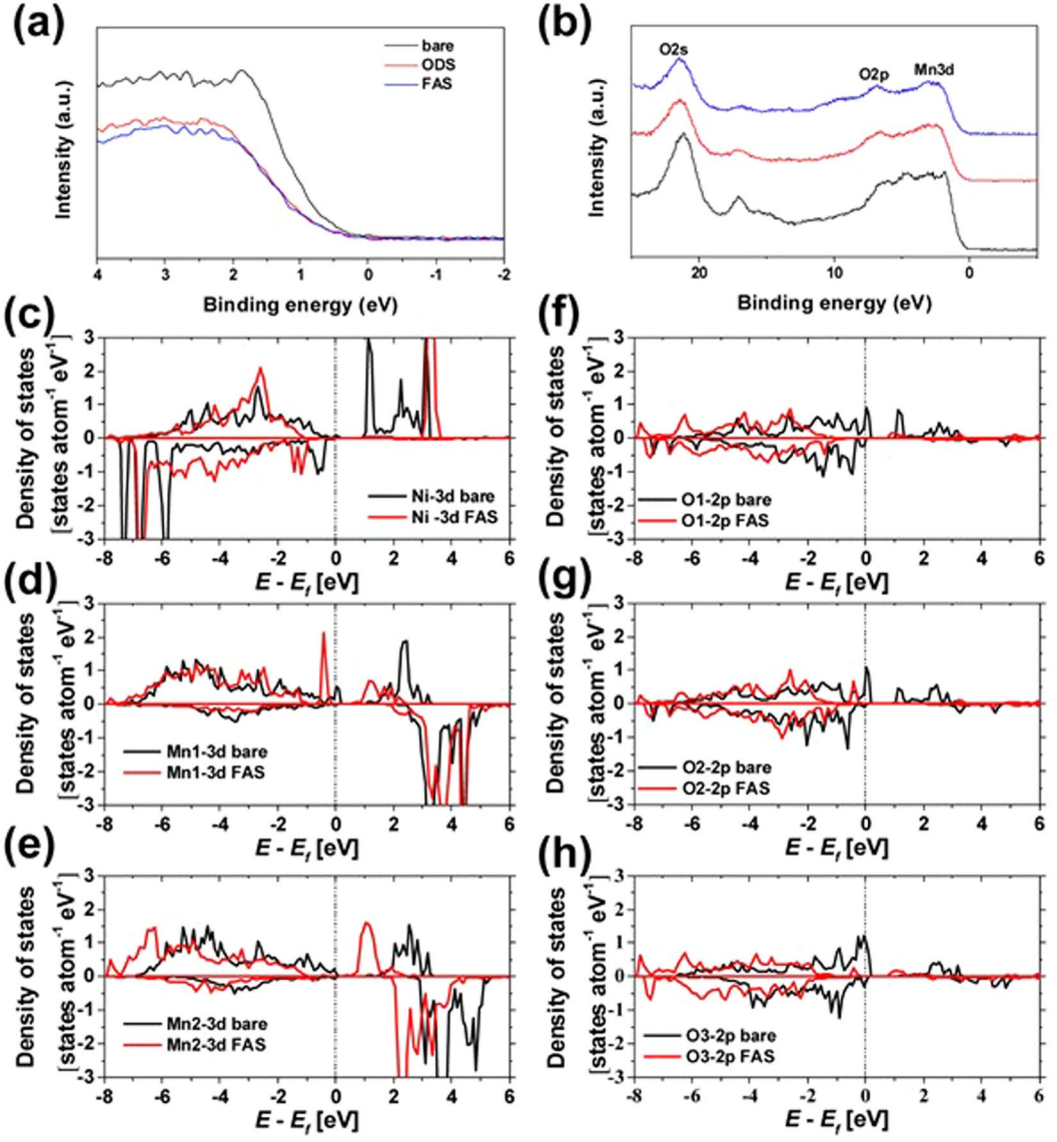
Impacts of the SAM coatings on the electronic structures of the FAS17 immobilized on the LNMO_4-*δ*_ electrode surface. (**a,b**) XPS valence band spectra of the fresh LNMO_4-*δ*_ electrodes with various SAM coatings and PDOS profiles obtained for the (**c**) Ni-3d, (**d,e**) Mn-3d, and (**f,h**) O-2p bands of the bare and FAS-coated LNMO_4-*δ*_ electrodes.

The broad C 1s XPS core-level spectra of the anodes depicted in Fig. 3f indicate the presence of graphitic C–C, C–O/C=O, hydrocarbon (–CH), lithiated carbon, and –CO_3_ species. The chemical environments of the graphite anodes were drastically changed after cycling. In particular, the relative peak areas of graphitic C–C bonds decreased after cycling regardless of the SAM composition (as compared with that of the fresh graphite electrode), indicating that an SEI layer was sequentially formed on the graphite electrode surface during cycling. It should be noted that the bare LNMO/graphite cell exhibited the lowest value C–C bonding ratio, suggesting that the thickest SEI layer was formed on the electrode surface (which can explain the observed slow Li-ion transport at the graphite anode/electrolyte interface). Furthermore, additional XPS peaks due to the presence of –CO_3_ species were clearly detected for the bare LNMO/graphite cells after cycling, but not for the SAM-coated LNMO_4-*δ*_/graphite cells, which was consistent with the trends observed for the O 1s XPS spectra discussed earlier.

EIS measurements were performed for both the anodes and cathodes extracted from the full cells to further investigate the positive impacts of the SAM coatings on the kinetic parameters of the battery reactions (Figure S4). The LNMO_4-*δ*_ electrodes immobilized with the FAS17 monolayers exhibited the lowest resistance, which was characterized by the two semicircles in the high- and low-frequency ranges denoted as *R*_sf_, and *R*_ct_, respectively. In contrast, the *R*_ct_ of the bare LNMO_4-*δ*_ electrode significantly increased after cycling due to the degradation of the LNMO_4-*δ*_ crystal surface primarily caused by the formation of LiF and metalfluoride species, leading to the impedance growth. Interestingly, the surface modification with the ODS monolayer also produced a positive effect on the impedance growth; however, it was not as significant as that of the FAS17 monolayer.

Similar to the cathode side, the bare LNMO_4-*δ*_/graphite cell exhibited the highest resistance in the anode side (Figure S4b); the obtained kinetic parameters are summarized in Table S1. Interestingly, the SAM coating on the cathode surface greatly contributed to the reduction of *R*_sf_ at the anode surface, as compared to the values obtained for the bare LNMO_4-*δ*_ cells. It can be suggested that the SAM coating of the cathode inhibits the excessive growth of the SEI layer on the anode surface, which is driven by the reduction of the oxidized electrolyte species (such as *β*-diketones coordinated with the Mn ions generated near the LNMO_4-*δ*_ electrode surface, reported by Jarry *et al*.^26^).

Meanwhile, the results of EIS measurements conducted for the pristine LNMO electrodes at various temperatures demonstrated that organosilane molecules could be used as effective surface modifiers for resolving the issues related to the slow Li^+^ transport kinetics at the electrode/electrolyte interface. This is one of strong merit in the SAM system, as comparing to use other surface coating approaches^13–21^. In LIBs, the rapid Li-ion transport from the cathode to the anode is essential for achieving the high rate performance during the charge–discharge process. The latter can be realized via several reactions, including the desolvation and subsequent intercalation of Li ions at the electrode/electrolyte interface^27–30^. The conductivities of the cells (determined from the corresponding Nyquist plots) were plotted at various temperatures in accordance with the Arrhenius law (Figures S5). The activation energies of the bare, ODS-coated, and FAS-coated cathodes estimated from the slopes of these plots were 0.69, 0.62, and 0.59 eV, respectively. Since this trend is consistent with the measured surface film resistances after cycling, it can be concluded that the deposition of a homogeneous organo-silane monolayer facilitates the Li^+^ transport at the electrode/electrolyte interface. Note that the presence of fluorocarbon chains on the cathode surface reduced its activation energy (as compared to that of the cathode modified with hydrocarbon chains). This phenomenon can be explained by the intrinsic properties of highly oriented fluorocarbon chains such as high electronegativity, good dielectric properties, and peculiar soluble properties.

Because the degradation of the LNMO_4-*δ*_ electrode was caused by the dissolution of Mn ions and gas evolution, its stabilization mechanism involving the passivation of M–M bonds and O–O bonds on the surface of active materials should be elucidated for the utilized modifiers. Hence, in this work, XPS valence band spectroscopy measurements and DFT calculations were performed to determine the electronic structure of the FAS monolayer immobilized on the LNMO_4-*δ*_ electrode surface. Figures 3a,b shows the XPS valence band spectra of the functionalized LNMO_4-*δ*_ electrodes with different binding energy ranges. The valence band edge positions apparently shifted to lower levels, while the corresponding peak areas decreased due to the SAM immobilization regardless of the molecular structure. Furthermore, SAM coating promoted the overall shifts of the positions of the valence band peaks to lower energies, including the Mn 3d, O 2p, and O 2s XPS spectra. Since a similar shift of the valence band edge position was observed for the LNMO_4-*δ*_ crystals containing F^–^ ions (LiNi_0.5_Mn_1.5_O_4–x_F_x_)^31^ with good high-voltage durability (Figure S6), both the ODS and FAS17 molecules passivated the Mn–Mn and O–O bonds located on the top surface of LNMO_4-δ_ crystals, thus preventing gas evolution, Mn^3+^ dissolution, and the formation of an excessive SEI layer.

To verify the observed shift of the valence band edge to lower energies by the immobilization of the FAS17 monolayer, DFT calculations were performed to determine the partial densities of states (PDOSs) of Ni (4b), Mn (12d), and O (8c, 24e) atoms in the vicinity of the Si atoms of the FAS17 molecules attached to the vacancy 16c  sites in the outermost surface atomic layer of the (111) face of the LNMO crystal. In these calculation, the stoichiometric LNMO {111} face model originally developed by the Persson group has been utilized^32^. In this model, the formation of Li^+^/Mn^2+^ antisite defects occupying the 8c sites of the second top layer was observed, thereby simultaneously achieving the stoichiometric composition and minimum surface energy. Figure 3c–h shows the changes in the PDOS profiles obtained for the Ni-2d, Mn-2d, and O-2p bands (the corresponding Ni, Mn1, Mn2, O1, O2, and O3 sites are indicated by the arrows in Figure S7) accompanying the immobilization of FAS17 molecules. It was found that all PDOS peaks located near the Fermi level were shifted to lower energies after immobilization, which was consistent with the results of XPS valence band measurements. The immobilization of FAS17 molecules stabilizes the LNMO crystal surface through the passivation of its M–M and O–O bonds and suppression of the side reactions at the LNMO_4-*δ*_/electrolyte interface. Note that new sharp peaks appeared below the Fermi level in the Mn2 band after the modification with the FAS17 monolayer, indicating that the manganese ions on the LNMO surface were reduced from the +4 to the +3 state due to the formation of Si–O–Mn bonding. Furthermore, the electron spin states (including the up and down ones) were not equally distributed across the initial LNMO (111) faces in the utilized model. The changes in the calculated Bader charges of each atoms were summarized in Table S2. The Bader charge is an integrated charge density bounded by zero flux surfaces on which the charge density is a minimum perpendicular to the charge density surface. The obtained charges for Mn and O were smaller and larger than the nominal charges, respectively. It may be related to the contribution of the covalent bonding nature. Since all these anomalous characteristics disappeared after the surface modification with FAS17 molecules, it can be concluded that the surface Mn–Mn bonds were stabilized, leading to suppress the dissolution into electrolyte solution.

**Figure 4:**
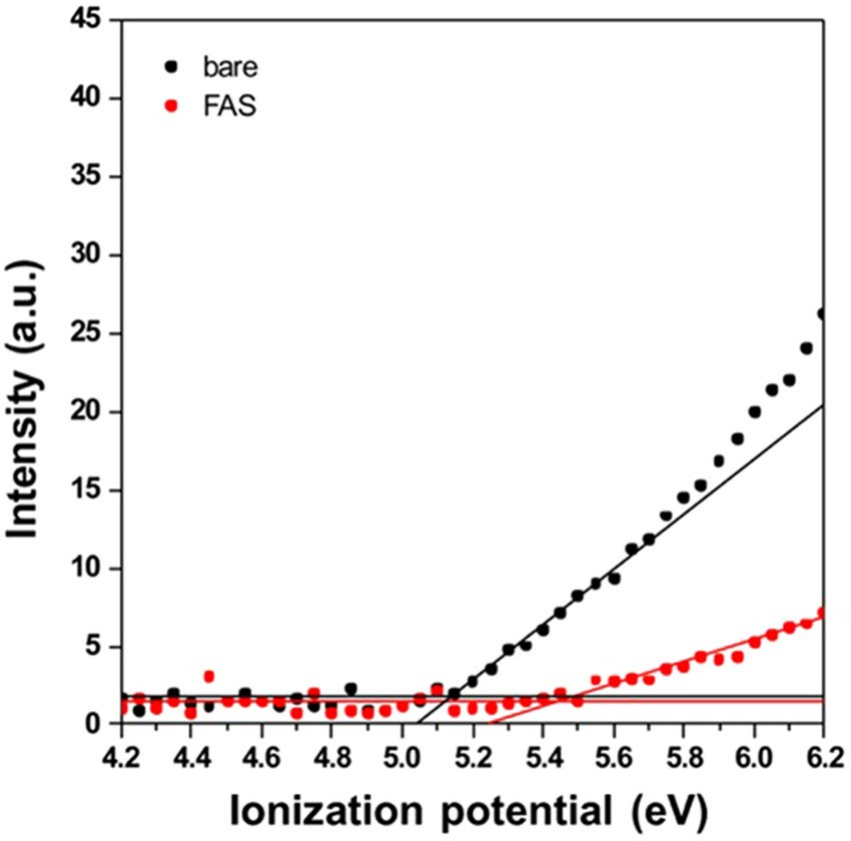
The impacts of the FAS17 modification on the ionization potential of the LNMO_4-*δ*_ electrodes.

In addition, ultraviolet photoelectron (UPS) measurements were performed to elucidate the effect of the FAS17 modification of the cathode on its ionization potential. As shown in Fig. 4, the threshold of the ultraviolet light energy required for the generation of a photoelectron from the LNMO_4-*δ*_ electrode surface increased, and the number of produced photoelectrons decreased after FAS17 coating. The observed spectral characteristics strongly suggest that the formation of Si–O–Mn bonding via the immobilization of FAS17 molecules increases the energy difference between the Fermi level and the vacuum level at the LNMO_4-*δ*_ electrode surface and that the presence of FAS17 molecules significantly decreases the probability of the reduction of both Ni^2+^ and Mn^3+^ ions due to the oxidative decomposition of the electrolyte at a high voltage. The obtained experimental results show that the SAM coating of the electrode surface acted as a homogeneous and dense physical buffer layer, which modified and stabilized the electrode surface and positively affected the electrochemical reactions occurring at the electrode/electrolyte interface.

**Figure 5:**
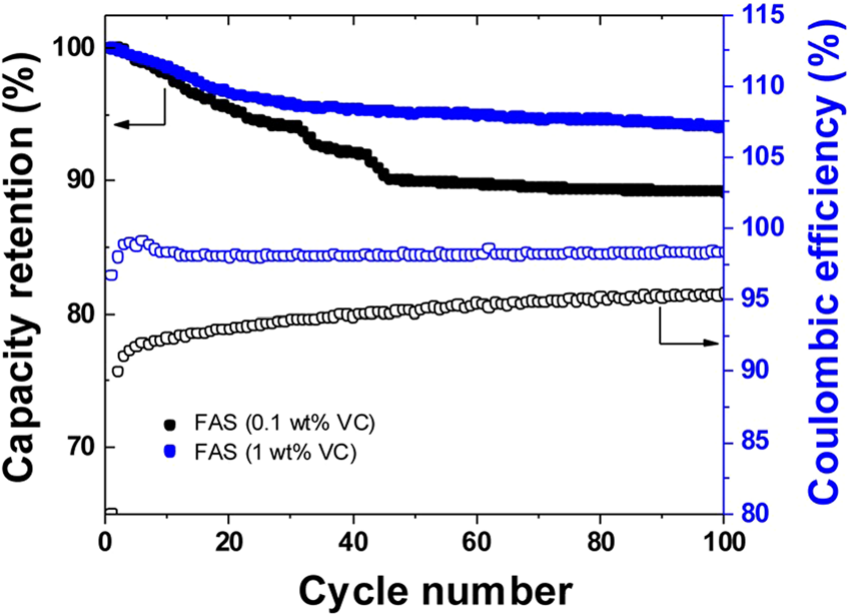
Capacity retention and variations of the Coulombic efficiencies of the FAS-coated LNMO_4-*δ*_/graphite cells with 0.1 and 1.0 wt% of VC at a charge-discharge rate of 1 C.

Finally, the cycling tests were performed again at room temperature and charge-discharge rate of 1 C for 100 cycles for the FAS17-coated LNMO_4-*δ*_/graphite cells containing 1.0 wt.% VC (Fig. 5). We found surface passivation with FAS17 molecule provides additional benefit in the high voltage durability through the stabilization of graphite anode by using VC additive. The capacity retentions of the FAS17-coated samples was significantly enhanced to 95% after cycling. Furthermore, the full cell exhibited high Coulombic efficiency of around 98% after 100 cycles, and its change as a function of cycles gently continue to be flat as comparing to that case of 0.1wt.% VC containing cells. Organic coating strategies based on additives of small molecules in electrolytes are not entirely new in the chemistry of lithium ion batteries. However, these approaches have highly limited performance enhancement due to the electrochemical dilemma driven by HOMO-LUMO analogies on the oxidative and reductive decomposition of electrolytes appeared at the cathode and anode surface. For example, it is known that VC molecules as an typical additives which shows positive effect for anode side, represents negative effects at the cathode surface due to its lower durability to oxidation. Choi el al demonstrated interesting polymeric electrolyte coating technique. The stabilization LNMO surface via the chelation between Mn^3+^ and polymeric electrolytes retained original capacity after cycles^21^. In contrast to our system, for instance, the capacity retention using full cell with carbon anode was maximally 91.5% at room temperature after 100 th cycles, and which it was a little lower than our results (>98%). We believe the coating strategies which are confronted with a HOMO-LUMO dilemma have a limitation in the enhancement of high voltage durability. Furthermore, the polymeric electrolyte system showed the C rate capability degradation due to the impedance growth after cycling. Meaning that it hardly control the oxidative decomposition of electrolytes and additives in the 5V-system, in contrast to the 4 V systems due to different chemistries and side parasitic reactions. We believe that the stabilization of cathode surface via directly tuning the ionic potential (LUMO level engineering), driven by modification with FAS molecule provides new aspects toward to push the above limitations.

## Discussion

The effects of the ultra-thin organo-silane monolayer immobilized on the oxygen-deficient spinel LNMO cathodes on their electrical and electrochemical characteristics were investigated for the first time in this study by various experimental and computational approaches. The obtained results indicate that the deposited SAM coatings reduced the activation barrier for the interfacial Li ion transfer and stabilized Mn^3+^ ions near the surface, which positively affected the electrochemical reactions occurring at the electrode/electrolyte interface. Hence, the surface stabilization with fluorocarbon-containing organo-silane coatings represents a new promising direction for the development of high-voltage cathode materials.

## Method

### Preparation and characterization of SAM-coated LNMO electrode

LiNi_0.5_Mn_1.5_O_4-*δ*_ (LNMO) crystals were grown from a LiCl–KCl mixed flux^22,31^. Self-assembled monolayers were formed on the surface of LNMO crystals via the vapor phase deposition conducted at a temperature of 150 °C and atmospheric pressure^22^. Fluoroalkylsilane (FAS17: F_3_C(CF_2_)_7_(CH_2_)_3_Si(OCH_3_)_3_ and octadecylsilane (ODS: H_3_C(CH_2_)_17_Si(OCH_3_)_3_) were used as precursor molecules for SAM coatings. Although liquid-phase processing is typically used for SAM preparation, the specified vapor-phase process was utilized in this study because it was expected to reduce the deposition of aggregated organosilane molecules, which tended to degrade the quality of the produced SAMs. The porous cathodes based on LNMO crystals were prepared by a conventional pasting technique at a mixing ratio of LNMO, acetylene black, and polyvinylidene fluoride equal to 90:5:5 (w/w). The obtained mixture was diluted with *N*-methyl-2-pyrrolidone, and the viscosity of the resulting paste was adjusted to 5.1 Pa·s. The prepared paste was coated on the surface of Al foil with a thickness of 20 μm using an applicator, after which the produced electrode was dried at a temperature of 120 °C for 12 h in vacuum. The electrode density was adjusted to 3 g/cm^3^. Galvanostatic charge–discharge profiles were recorded using a coin-type R2032 cell. Li metal foil (Honjo Metal Co., Ltd) and graphite-based composite electrode supplied by Hitachi Maxell Corp. were used as the counter electrodes for the half-cell and full cell, respectively. A porous polypropylene film purchased from Celgard was used as a separator (#2500). A solution of 1 M of LiPF_6_ in a mixture of EC and DMC (3:7 v/v) was used as the electrolyte. The coin-type cell was assembled and disassembled inside an Ar-filled glovebox (MIWA MFG Co. Ltd) with a controlled level of H_2_O not exceeding 1 ppm. X-ray photoelectron core-level and valence band spectroscopies (XPS; JPS-0910, JEOL) with a monochromic Al-Kα source operated at a voltage of 15 kV and current of 15 mA were used for the evaluation of the SAM coating effects on the high-voltage durability of the studied cells. Photoelectron work functions of the LNMO electrodes were evaluated using a photoelectron spectrometer (AC-2, RIKEN KEIKI). Galvanostatic charge–discharge tests were conducted in the cut-off voltage range from 3.5 to 4.8 V (vs Li^+^/Li) using a battery test unit (HJ1001SD8, HOKUTO DENKO). Electrochemical impedance spectroscopy (EIS) measurements were performed in the frequency range between 2 MHz and 1 mHz using a VSP-300 electrochemical workstation (Bio-logic).

### Computational methods

Vienna *ab initio* simulation package^33,34^ with the generalized gradie nt approximation (GGA − PBEsol) + *U*^35^ and projector-augmented wave methods was used in this study^36^. For the GGA + *U* calculations, the *U* values of the *d*-orbitals of Ni and Mn were set to 6.0 and 3.9 eV, respectively, in accordance with the results of previous studies^37–39^. An energy cut-off of 500 eV and a 3 × 3 × 3 k-point mesh were used for bulk calculations, while Γ point k-point sampling was utilized for slab calculations. A superstructure containing 56 atoms in the cubic spinel lattice of 8(LiNi_0.5_Mn_1.5_O_4_) was used for the bulk calculations. The relaxation of the crystal structure was allowed for the bulk models. The final energies of the optimized structural geometries were recalculated to correct for the changes in the plane-wave basis induced by the relaxation (the atoms on the LNMO surface were arranged manually while retaining their crystallographic symmetry and chemical stoichiometry). The crystallographic symmetry of the top and bottom slab surfaces is essential for making a rational computational prediction. Therefore, Si-containing FAS17 species were placed at the same crystallographic sites of both the top and bottom slab surfaces. The slab cells with fixed lattice parameters were constructed from the relaxed bulk structure. The atomic positions of the two surface layers of Ni/MnO_6_ octahedra connected to FAS17 molecules were relaxed, while the geometries of other bulk regions of the slab remained fixed. To simulate the surface facets with vacuum thicknesses of 20 Å, slabs with thicknesses greater than 20 Å were utilized.

## Electronic supplementary material

Supplementary Information
