# Supplementary material for: New Insight for Surface Chemistries in Ultra-thin Self-assembled Monolayers Modified High-voltage Spinel Cathodes
Source: Sci Rep. 2018 Aug 6;8:11771. doi: 10.1038/s41598-018-30135-z (PMC6079065; doi:10.1038/s41598-018-30135-z)

Supporting information

New Insight for Surface Chemistries in Ultra-thin Self-assembled Monolayers Modified High-voltage Spinel Cathodes

Dae-wook Kim[1,3], Shuhei Uchida[1,3], Hiromasa Shiiba[1], Nobuyuki Zettsu*[,1,2] and Katsuya Teshima*[1,2]

[1]Department of Materials Chemistry, Faculty of Engineering, Shinshu University, 4-17-1 Wakasato, Nagano 380-8553, Japan

[2]Center for Energy & Environmental Science, Shinshu University, 4-17-1 Wakasato, Nagano 380-8553, Japan

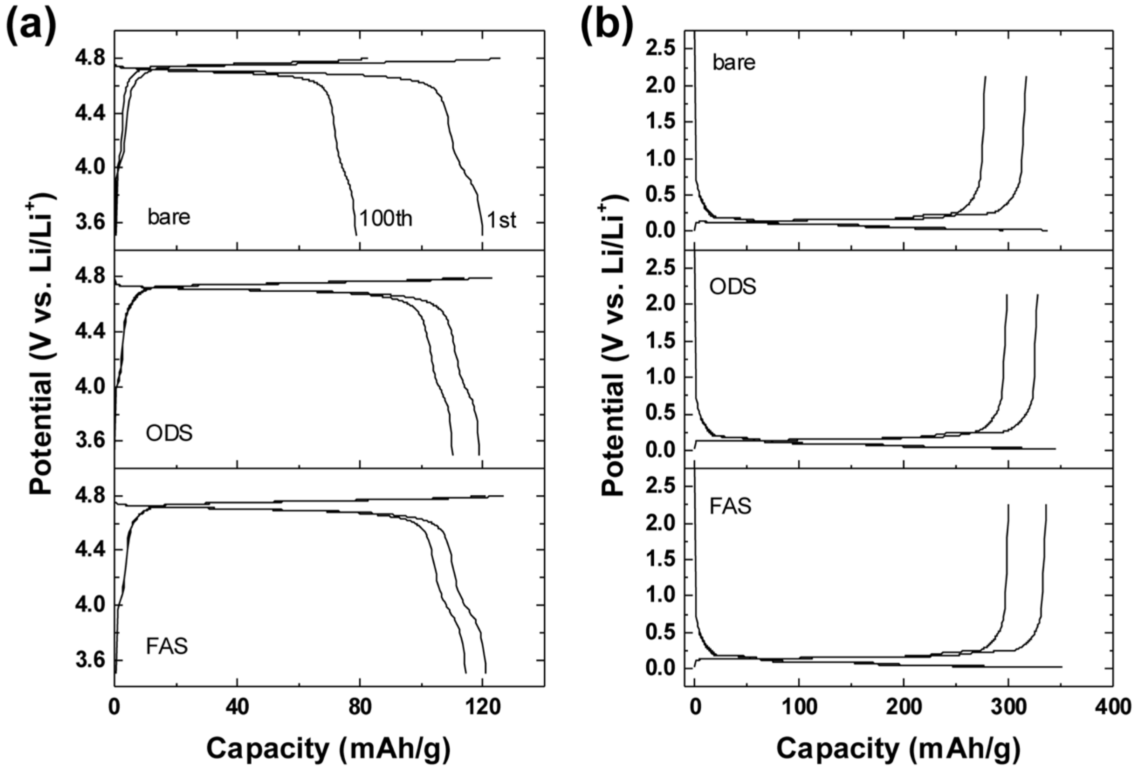

Figure S1 The initial and 100th galvanostatic charge–discharge profiles. The SAM-coated $LNMO_{4-\delta}$/graphite cells obtained for the (a) cathode and (b) anode at a charge-discharge rate of 0.2 C using the three-electrode cell.

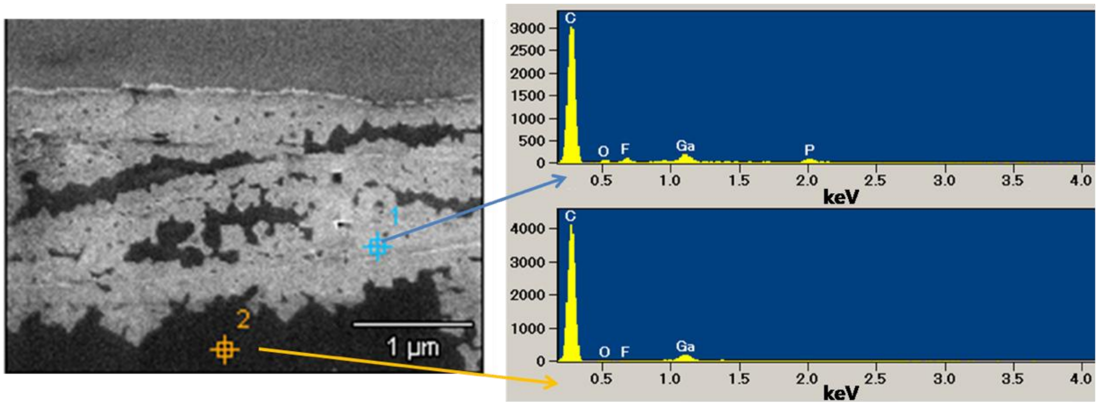

Figure S2 EDS spectrum of the sliced graphite anode extracted from the cycled bare $LNMO_{4-\delta}$/graphite cell.

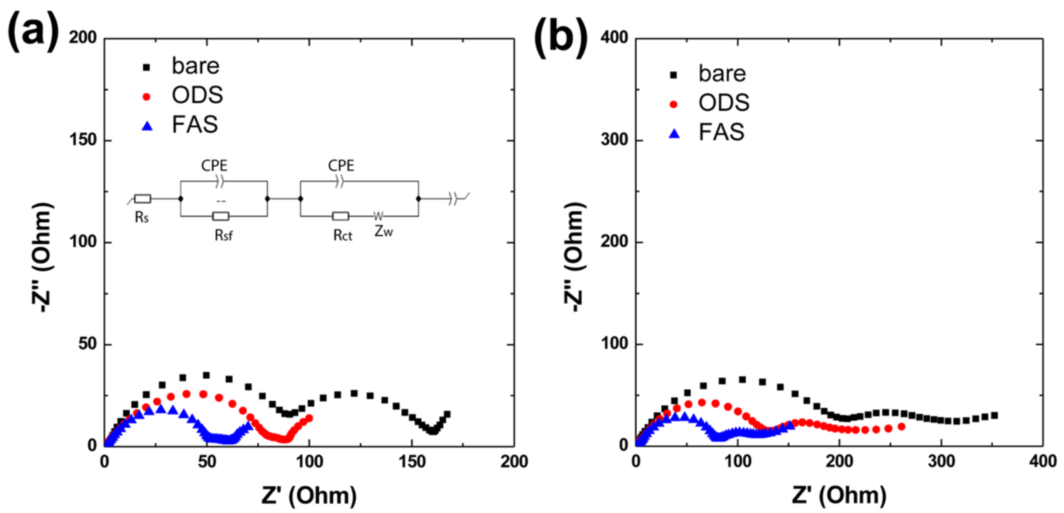

Figure S3 EIS spectra of (a) various SAM-coated $LNMO_{4-\delta}$ cathodes and (b) the corresponding graphite anodes after 100 cycles. All experiments were performed by using reassembled into Li half cells which were constructed with the electrodes extracted from the full cells after 100 cycles.

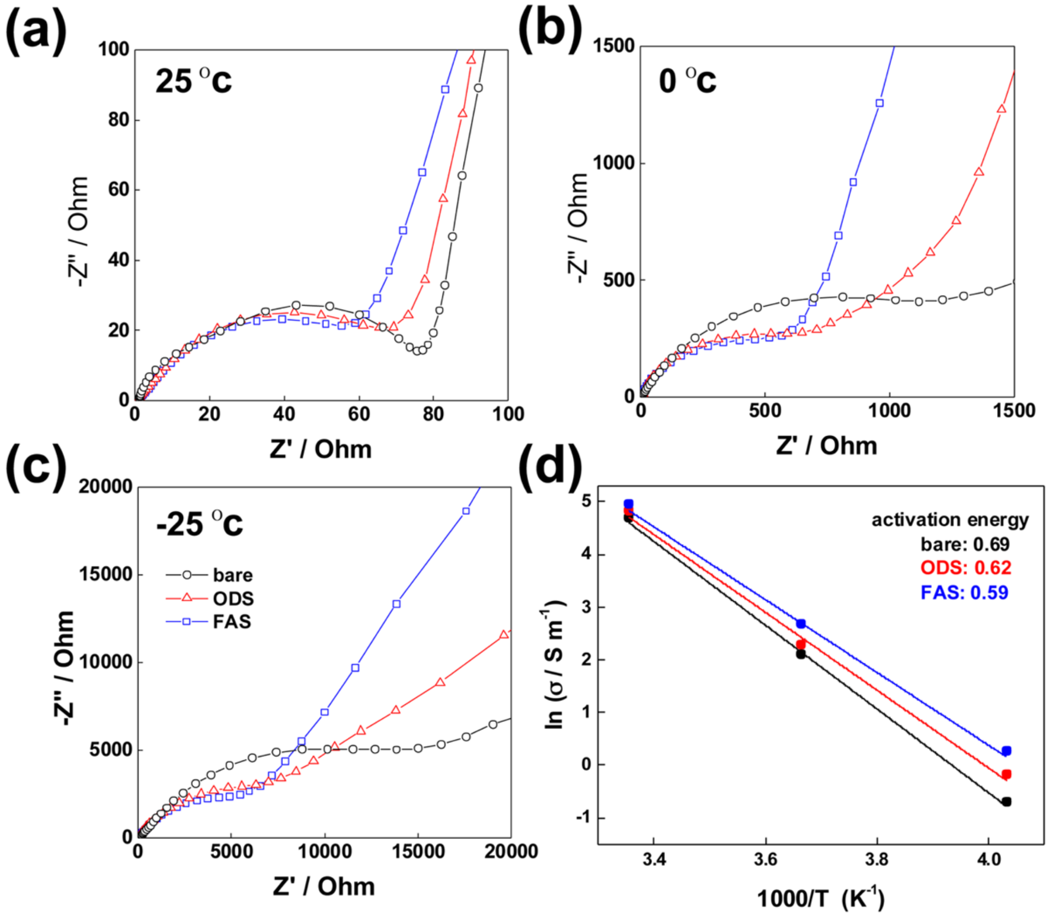

Figure S4 Activation energy of SAM-coated $LNMO_{4-\delta}$/Li cell. (a–c) Nyquist plots constructed for the SAM-coated LNMO/Li cells at various temperatures. (d) Arrhenius plots constructed for the interfacial resistivity of the $LNMO_{4-\delta}$-based half-cells with various SAM coatings.

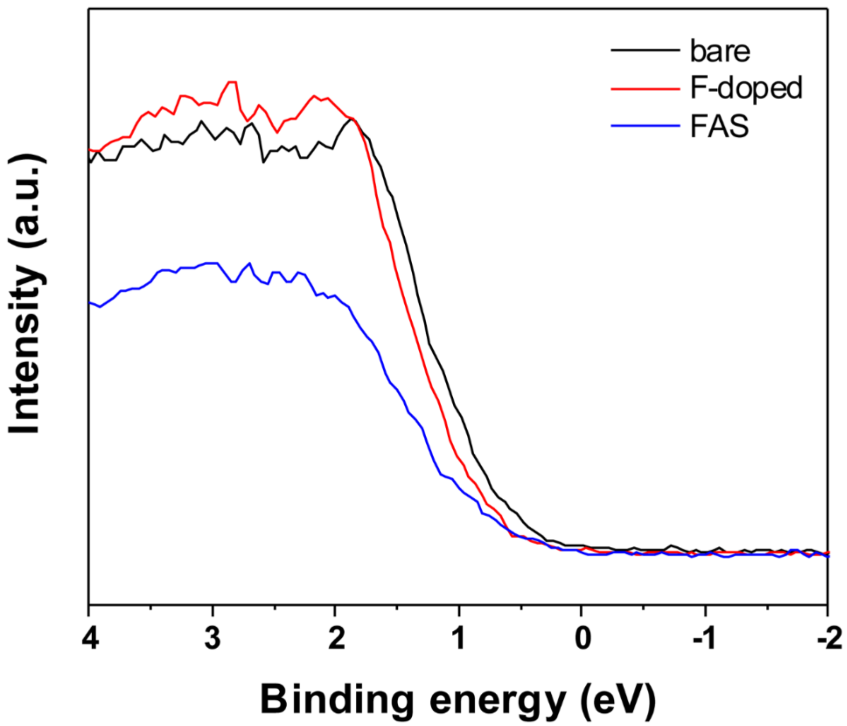

Figure S5 XPS valence band spectra of the $LNMO_{4-\delta}$ and F-substituted $LNMO_{4-\delta}$ crystals.

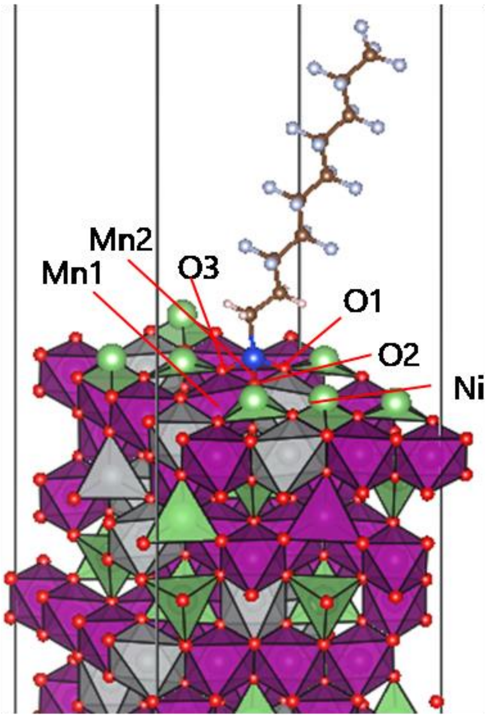

Figure S6 DFT simulations model of the LNMO {111} face immobilized with a single FAS molecule.

Table S1. Kinetic parameters of the SAM-coated LNMO/graphite cells obtained before and after cycling test.

	before cycling		after cycling			
	full-cell		cathode		anode	
	R_{sf} (Ω)	R_{ct} (Ω)	R_{sf} (Ω)	R_{ct} (Ω)	R_{sf} (Ω)	R_{ct} (Ω)
bare	12.36	14.32	90.48	63.74	193.40	126.70
ODS	9.416	23.60	81.11	13.36	136.70	76.36
FAS	10.71	20.17	51.91	7.59	84.47	34.95

Table S2. The calculated Bader charges of each atoms on the model of the bare and single FAS molecule functionalized LNMO {111} faces.

	Bader charge				
	Mn1	Mn2	O1	O2	O3
Bare LNMO	2.98 (+2.11)	3.11 (+2.10)	-0.49 (-1.02)	-0.42 (-1.02)	-0.12 (-1.11)
FAS17/LNMO	3.86 (+1.90)	3.10 (+2.09)	-0.05 (-1.73)	0.01 (-1.80)	0 (-1.71)

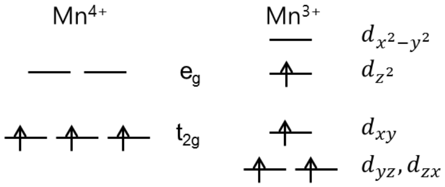

Supplement: Supplementary file 1 — Supplementary Information [file 41598_2018_30135_MOESM1_ESM.pdf]
